# Mitochondrial reactive oxygen species (ROS) as signaling molecules of intracellular pathways triggered by the cardiac renin-angiotensin II-aldosterone system (RAAS)

**DOI:** 10.3389/fphys.2013.00126

**Published:** 2013-05-30

**Authors:** V. C. De Giusti, C. I. Caldiz, I. L. Ennis, N. G. Pérez, H. E. Cingolani, E. A. Aiello

**Affiliations:** Facultad de Ciencias Médicas, Centro de Investigaciones Cardiovasculares, UNLP-CONICETLa Plata, Argentina

**Keywords:** cardiac myocyte, second messenger systems, sodium-hydrogen antiporter, sodium-bicarbonate symporters, reactive oxygen species

## Abstract

Mitochondria represent major sources of basal reactive oxygen species (ROS) production of the cardiomyocyte. The role of ROS as signaling molecules that mediate different intracellular pathways has gained increasing interest among physiologists in the last years. In our lab, we have been studying the participation of mitochondrial ROS in the intracellular pathways triggered by the renin-angiotensin II-aldosterone system (RAAS) in the myocardium during the past few years. We have demonstrated that acute activation of cardiac RAAS induces mitochondrial ATP-dependent potassium channel (mitoK_ATP_) opening with the consequent enhanced production of mitochondrial ROS. These oxidant molecules, in turn, activate membrane transporters, as sodium/hydrogen exchanger (NHE-1) and sodium/bicarbonate cotransporter (NBC) via the stimulation of the ROS-sensitive MAPK cascade. The stimulation of such effectors leads to an increase in cardiac contractility. In addition, it is feasible to suggest that a sustained enhanced production of mitochondrial ROS induced by chronic cardiac RAAS, and hence, chronic NHE-1 and NBC stimulation, would also result in the development of cardiac hypertrophy.

## Introduction

The renin-angiotensin-aldosterone-system (RAAS) represents one of the main endocrine systems that regulate cardiac physiology. At present, it is well recognized that angiotensin II (Ang II) is produced and secreted locally in several tissues, including the heart (Husain et al., 1994). Sadoshima's group has shown that the hormone is secreted from intracellular vacuoles in response to myocyte stretching for the first time. This Ang II exerts autocrine and paracrine effects, leading to cardiac hypertrophy (Sadoshima et al., 1993; Sadoshima and Izumo, 1996). Cingolani's group conducted an in depth study of this autocrine pathway as a physiological mechanism responsible for the slow force response (SFR) to myocardial stretch (Cingolani et al., 2001, 2003) and showed the similarities of both the physiological and pathological pathways (Cingolani et al., 2008). The critical role played by the cardiac Na^+^/H^+^ exchanger (NHE-1) activation in both physiological and pathological responses was demonstrated not only pharmacologically with NHE-1 inhibitors (Cingolani et al., 2011) but also by specific NHE-1 silencing following direct intramyocardial injection of small interfering RNA into rat left ventricular wall (Morgan et al., 2011; Cingolani et al., 2013). The precise mechanism to explain pathological responses is unclear and warrants further investigation. However, it is possible that the time of exposure to the stimulus and the amount of ROS produced could be important in determining the physiological or pathological pathways. Increasing time and amount of ROS exposure could exert a differential impact in calcium handling, an initial acute response leading to inotropic effects followed by a sustained response that could involve calcium-activated targets that participate in cardiac hypertrophy or heart failure, like calcineurin, or Ca^2+^-calmodulin-dependent kinase type II (CaMKII).

Although still somewhat controversial (Silvestre et al., 1998, 1999; Takeda et al., 2000; Gomez-Sanchez et al., 2004; Chai and Danser, 2006), it has been suggested that aldosterone synthase exists in the myocyte (Silvestre et al., 1998, 1999; Takeda et al., 2000), supporting the presence of a local RAAS (Varagic and Frohlich, 2002). Furthermore, the link between Ang II or its AT_1_ receptor, and the mineralocorticoid receptor (MR) is an accepted fact (Lemarie et al., 2008; Grossmann and Gekle, 2009). Consistently, it has also been described that some physiological cardiac effects of Ang II, as the SFR, can be prevented in the presence of MR blockers (Caldiz et al., 2011).

Although the idea that mitochondria are the main sources of basal reactive oxygen species (ROS) in other mammalian cells has been recently challenged, (Brown and Borutaite, 2012) their role as a very important source of ROS in the heart has been widely accepted. Mitochondrial superoxide anion (O_2_^−^) and its product, hydrogen peroxide (H_2_O_2_), were demonstrated to be important molecules implicated in several cardiac functions usually acting as second signal molecules of RAAS (Kimura et al., 2005a,b; Caldiz et al., 2007, 2011; De Giusti et al., 2008, 2009).

In this review, we will briefly summarize the current knowledge about the involvement of mitochondrial ROS as mediators of the signaling pathways triggered by RAAS in cardiac myocytes without stressing out if they participate in acute or chronic signals. We will discuss the participation of the different components of RAAS in ROS production and in cardiac signaling leading to physiological and pathological responses. Particularly, we will remark the implication of the ion transporters (NHE-1 and NBC) in sodium and calcium overload and its relation with ROS signaling.

## Angiotensin II, endothelin-1, aldosterone and epidermal growth factor: independent signals or different components of the same cardiac system?

Ang II is involved in the regulation of almost all cardiac functions. At present, it is well known that Ang II stimulates membrane ions transporters as NHE-1 (Fliegel and Karmazyn, 2004; Cingolani et al., 2005) and Na^+^/HCO_3_^−^ cotransporter (NBC) (Baetz et al., 2002; De Giusti et al., 2009; Aiello and De Giusti, 2012). These regulations are crucial for the correct electrical and mechanical cardiac functions. On the other hand, it is important to keep in mind that when RAAS is chronically active it is responsible for several cardiac diseases, for example, hypertrophy, heart failure and electrical disturbances (Domenighetti et al., 2007; Fischer et al., 2007; Mehta and Griendling, 2007; Palomeque et al., 2009; Li et al., 2013).

The mechanism of how the activation of NHE-1 or NBC regulates cardiac contractility seems to involve the increase in intracellular Na^+^ concentration ([Na^+^]_i_) (Vaughan-Jones et al., 2006) due to the activation of these transporters and the subsequent increase in intracellular calcium concentration ([Ca^2+^]_i_) due to the activation of the reverse mode of the Na^+^/Ca^2+^ exchanger (NCX) (Perez et al., 2001; Rothstein et al., 2002; Bril, 2003; Morgan et al., 2011). Interestingly, the same pathway is proposed to explain the development of cardiac hypertrophy (Ennis et al., 2007; Cingolani et al., 2008).

In addition, endothelin-1 (ET-1) and aldosterone (Ald) are key modulators of cardiac physiology *per se*. We have shown that ET-1 activates the NHE-1 (Aiello et al., 2005; De Giusti et al., 2008) leading to a positive inotropic effect (Szokodi et al., 2008). Moreover, Ald has been shown to activate NHE-1, (De Giusti et al., 2011) increase NHE-1 expression (Karmazyn et al., 2003) and induce left ventricular hypertrophy independently from its classical effects on regulation of renal Na^+^ excretion and blood pressure (Qin et al., 2003; Yoshida et al., 2005; Diez, 2008). Classically, Ald enters the cells and binds to the MR located mainly in the cytosol. This binding translocates the MR to the nucleus, where it acts as a ligand-induced transcription factor. However, evidence has been presented that activated MR can elicit additional non-classical effects, which do not require transcription or translation of genes (Ebata et al., 1999; Mihailidou et al., 2004; Chai et al., 2005; Grossmann and Gekle, 2009). In addition, several of these rapid non-genomic effects of Ald involves the transactivation of the epidermal growth factor receptor (EGFR) (Grossmann and Gekle, 2007; Grossmann et al., 2007), which can, in turn, stimulate the NHE-1 (De Giusti et al., 2011). Moreover, it was reported that at least a small fraction of the classic MR is located in the cell plasma membrane where it is co-localized with the EGFR, inducing the transactivation of the latter (Grossmann et al., 2010).

More recently, it was demonstrated that certain non-genomic effects of Ald in vascular smooth muscle were due to simultaneous activation of MR and a surface membrane G protein–coupled receptor, the GPR30 (Gros et al., 2011, 2013). In agreement, growing evidence is appearing which demonstrate that GPR30 could be another Ald receptor involved in the rapid effects of the hormone in the cardiovascular system (Gros et al., 2011; Meyer et al., 2011).

**Figure 1:**
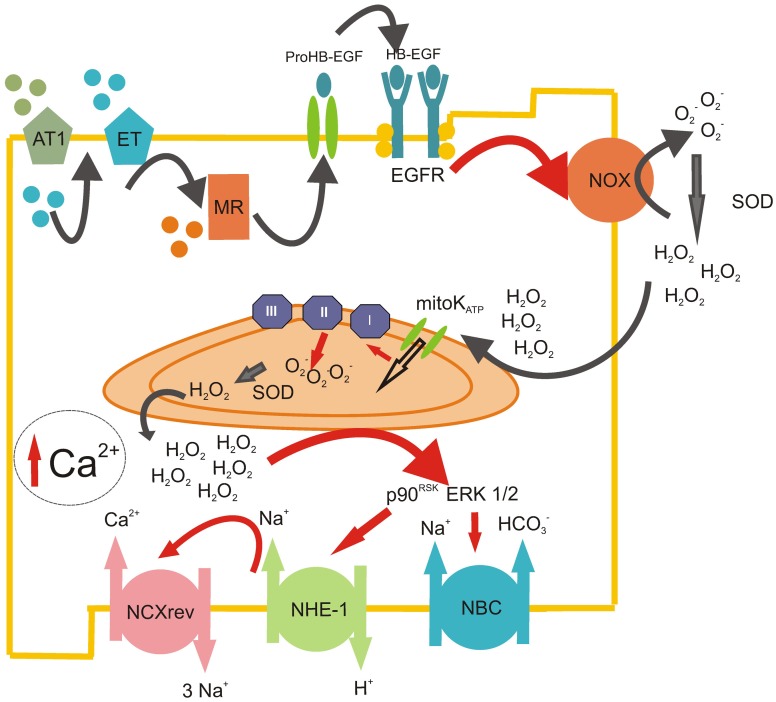
**ROS-induced ROS-release mechanism triggered by RAAS.** Scheme representing the sequential steps involved in the production of mitochondrial ROS after the initial Ang II stimulation. Ang II acting on AT_1_ receptors induces the release of intracellular ET-1, which, in turn, acts in an autocrine manner on ET_A_ receptors. This autocrine action leads to the activation of the mineralocorticoid receptor (MR), which induces the transactivation of the EGFR, possibly via the release of membrane heparin-bound EGF (HB-EGF). The stimulation of the EGFR leads to the activation of the NADPH oxidase (NOX), which produces superoxide anion (O_2_^−^) and quickly dismutate by superoxide dismutase (SOD) to hydrogen peroxide (H_2_O_2_). This permanent and stable oxidant molecule produces the opening of mitochondrial ATP-dependant potassium channels (mitoK_ATP_) with the subsequent enhanced production of mitochondrial O_2_^−^ by the electron transport chain (mainly by complex II). These mitochondrial ROS are released to the cytosol (ROS-induced ROS-release mechanism), where they stimulate redox sensitive MAPkinases ERK 1/2 and p90^RSK^, which, in turn, activate NHE-1 and NBC, pH regulation transporters that induce the increase in intracellular Na^+^. Finally, this cytosolic Na^+^ increase favors the operation of the reverse mode of NCX, promoting the influx of Ca^2+^ into the cell. The enhancement of intracellular Ca^2+^ in the cardiomyocyte could lead to a positive inotropic effect in the short term and/or the development of cardiac hypertrophy in a time-prolonged scenario.

At present, it is accepted that many effects initially believed to be mediated by Ang II, as the positive inotropic effect (PIE), and the increase in the SFR after myocardial stretching, are in fact attributable to the action of ET-1, which is released by Ang II (Perez et al., 2003; Cingolani et al., 2006, 2008; Villa-Abrille et al., 2006). Moreover, Ald appears to mediate some Ang II effects that participate in the same pathway of Ang II and ET-1 (Figure 1) (Xiao et al., 2004; Lemarie et al., 2008; Caldiz et al., 2011). Recently, it has been demonstrated that EGF is also implicated in cardiac physiology (De Giusti et al., 2011), and it has been described that the transactivation of the EGF receptor (EGFR) is involved in some RAAS effects (Shah and Catt, 2003; Zhai et al., 2006; De Giusti et al., 2011). We have suggested that all these extracellular and intracellular stimuli are pieces of the same signaling pathway (Figure 1). In this scenario, the activation of the MR takes place downstream from the Ang II/ET-1 receptors and upstream of the EGFR. The activation of EGFR triggers the intracellular ROS production, which leads to the stimulation of different kinases that finally activate the NHE-1 (Caldiz et al., 2011).

Interestingly, almost all the effects of these hormones involve ROS-mediated pathways (Zhang et al., 2001; Caldiz et al., 2007, 2011; Bartosz, 2009). In this regard, it is accepted that Ang II, (Giordano, 2005; Kimura et al., 2005b; De Giusti et al., 2009), ET-1 (De Giusti et al., 2008; Kubin et al., 2011), Ald (Hayashi et al., 2008; Caldiz et al., 2011) and EGF (De Giusti et al., 2011) can activate NADPH oxidase (NOX), which then, as further explained below, can stimulate mitochondrial ROS production and mediate the effects of such hormones (Figure 1). Therefore, it seems clear that RAAS effects are in close relationship with ROS generation, and in order to be able to modulate RAAS signaling, we should investigate the regulation of ROS production in detail.

## Major sources of ROS: NOX, mitochondria and their cross-talk

ROS have been considered deleterious agents for a long time. However, in the last years, evidence has emerged supporting their role as second messengers (D'autreaux and Toledano, 2007). Under physiological conditions, the production of ROS is highly restricted to specific subcellular sites. The major sources of ROS in the cardiomyocytes are NOX (Bedard and Krause, 2007) and the I, II and III complexes of the mitochondrial respiratory chain (Camara et al., 2010; Dedkova et al., 2013; Drose, 2013; Li et al., 2013; Wojtovich et al., 2013). In this regard, complex II is emerging as the major modulator of mitochondrial ROS production (Drose, 2013). Moreover, it was proposed that complex II can adopt different roles as a producer or modulator of mitochondrial ROS, depending on the substrate supply and the activities of the other respiratory chain complexes (Drose, 2013). The primary function of complex II is to maintain the reduced state of mitochondrial chain complexes (Wojtovich et al., 2013). Importantly, it was demonstrated that complex II, instead of complex I or III, is the major source of ROS during heart failure (Dedkova et al., 2013). On the other hand, it has been proposed that the production of “deleterious ROS” during reperfusion can be related to complex I, while the generation of “signaling ROS” during preconditioning occurs at complex II (Drose, 2013). Although the precise mechanism is not clear yet, the modulation of complex II seems to be cardioprotective during ischemic preconditioning (Wojtovich et al., 2013). These data, however, do not support previous results which suggested that the production of ROS induced after mitoK_ATP_ opening is accounted by complex I (Andrukhiv et al., 2006), and that ischemic preconditioning is mediated by ROS generated after the activation of these channels (Oldenburg et al., 2004).

At present it is accepted that NOX produce extracellular O_2_^−^, which dismutate to H_2_O_2_. Although it has been generally assumed that H_2_O_2_ diffuses back into the cell across the plasma membrane, recent evidence suggests that it might preferentially enter the cell through specific aquaporin channels (Bienert et al., 2007; Miller et al., 2010), providing a potential mechanism through which ROS signaling could be regulated. It is also accepted that matrix H_2_O_2_ permeates through the mitochondrial inner membrane after being produced by the action of Mn-SOD, which dismutates mitochondrial O_2_^−^. However, it is important to note that O_2_^−^ also permeates mitochondrial membrane through anion channels (Bedard and Krause, 2007) and hence could potentially act as a signaling molecule. The SFR, which represents an acute and physiological response triggered by RAAS activation, was reported to be due to H_2_O_2_ signaling (Caldiz et al., 2007). Moreover, experiments by Sabri et al. (1998) and Rothstein et al. (2002) indicated that H_2_O_2_ is the intracellular signal leading to the activation of kinases that phosphorylate the NHE-1. On the other hand, O_2_^−^- not H_2_O_2_ - was reported to be the signaling molecule in the ET-1-induced stimulation of cardiac L-type calcium channels (Zeng et al., 2008). Nevertheless, if mitochondrial H_2_O_2_ or O_2_^−^ could cause different responses to RAAS activation, i.e., acute versus chronic, it would be an interesting topic that deserves future investigation.

NOX and mitochondria are not totally independent sources of ROS, since recent evidence demonstrate the existence of a substantial interplay between both sources, such as activation of one leading to the activation of the other (Dikalov, 2011). In 2000, Zorov et al. (2000) published the first study describing the phenomenon called “ROS-induced ROS-release” by which a small amount of ROS triggers greater ROS production from the mitochondria. Five years later, Dr. Kimura's group proposed the Ang II-induced NOX stimulation as the generator of the small amount of ROS triggering mitochondrial ROS production (Kimura et al., 2005a) (Figure 1). It is important to note that this “ROS-induced ROS-release” mechanism is implicated in Ang II-mediated preconditioning.

The main link between both sources of ROS seems to be the mitochondrial ATP-dependent potassium channel (mitoK_ATP_). It was demonstrated that the opening of these channels is crucial to stimulate ROS production by the respiratory chain. Three phenomena were proposed to activate the mitochondrial respiratory chain and produce ROS: moderate matrix swelling, matrix alkalinization and inner membrane depolarization (Pain et al., 2000; Andrukhiv et al., 2006). A still unresolved issue is how the mitoK_ATP_ are opened. On the one hand, it has been reported that O_2_^−^ can directly stimulate the mitoK_ATP_, (Zhang et al., 2001, 2007) on the other hand, there is enough evidence that demonstrate the involvement of PKC as an activator of mitoK_ATP_ (Sato et al., 1998; Costa et al., 2006; Costa and Garlid, 2008). In addition, other studies have proposed that the cardioprotective effect of G_i_-coupled receptor agonists are due to EGFR transactivation and subsequent stimulation of the PI3K/Akt pathway, which lead to a PKG-mediated opening of mK_ATP_ channels and increased O_2_^−^ production (Krieg et al., 2002, 2003, 2004). These authors proposed that PI3K/Akt increase nitric oxide levels, which, in turn, stimulates the guanylate cyclase, augmenting cGMP content and activating PKG, inducing the opening of mitoK_ATP_ channels (Krieg et al., 2004; Oldenburg et al., 2004).

**Figure 2:**
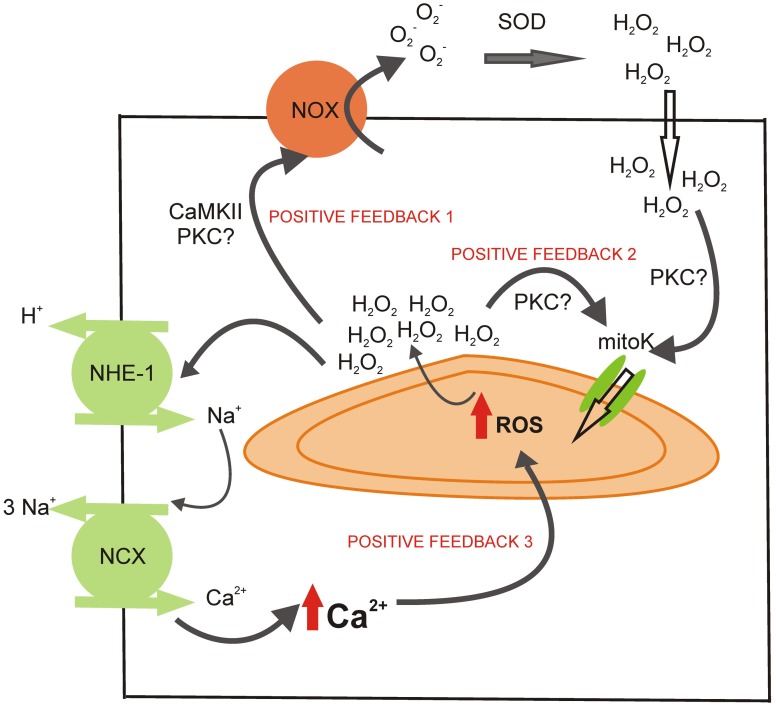
**Potential sites of positive feedback mechanisms involved in the mitochondrial ROS production during the activation of RAAS.** The H_2_O_2_ released by the mitochondria during the ROS-induced ROS-release mechanism could activate NOX (possibly via CaMKII or PKC activation), cycling the mitochondrial ROS production (positive feedback 1). The mitochondrial ROS could also help to maintain the opening of mitoK_ATP_ (positive feedback 2), perhaps through the activation of PKC. Finally, the intracellular Ca^2+^ augmentation after NHE-1 and NCX reverse mode stimulation could induce mitochondrial Ca^2+^ load and further ROS production (positive feedback 3).

Interestingly, it seems that not only NOX-derived ROS trigger mitochondrial ROS production, but also a small amount of mitochondrial ROS released to the cytosol could potentially further activate ROS-induced ROS-release in neighboring mitochondria (Costa and Garlid, 2008). In addition, mitochondrial ROS can stimulate NOX directly or mediated by PKC activation (Doughan et al., 2008; Wenzel et al., 2008; Camara et al., 2010; Dikalov, 2011). These different signaling regulations create a truly cross-talk between the major sources of ROS (Daiber, 2010). This issue is important because it converts the mitochondrion to a ROS-amplifier. Myocytes spend little energy to start the intracellular signaling and then the cycle helps to potentiate ROS-production (Figure 2).

## The mitochondrial ROS are the mediators of RAAS- induced NHE-1 and NBC regulation, or is it the opposite?

ROS-mediated activation of NHE-1 (Sabri et al., 1998; Snabaitis et al., 2002; Caldiz et al., 2007, 2011; De Giusti et al., 2008) and NBC (De Giusti et al., 2009; Aiello and De Giusti, 2012) has been reported to be due to redox sensitive kinase-mediated phosphorylation. In this regard, there is enough evidence supporting the notion that ROS favors the activation of ERK 1/2 and p90^RSK^ in neonatal and adult cardiomyocytes (Sabri et al., 1998; Rothstein et al., 2002).

As described above, the components of RAAS are well known activators of ROS production (Hanna et al., 2002; Seshiah et al., 2002; Kimura et al., 2005b; Doughan et al., 2008). Moreover, in our lab, we have investigated the pathway by which myocardial stretch sequentially stimulates ROS production, activates ERK 1/2 and p90^RSK^ and finally leads to the stimulation of NHE-1 (Caldiz et al., 2007, 2011; Villa-Abrille et al., 2010). We demonstrated that NHE-1 stimulation is responsible for the SFR in the acute phase. Thus, we proposed that NHE-1 stimulation is potentially responsible, in a more prolonged term, for chronic and pathological responses, such as the development of cardiac hypertrophy (Cingolani et al., 2008).

As Figure 1 shows, RAAS signaling leads to an increase in ROS production and subsequent activation of ERK 1/2 and p90^RSK^ kinases, which stimulate both transporters, NHE-1 and NBC. The stimulation of these transporters might lead to an increase in [Na^+^]_i_ (Vaughan-Jones et al., 2006), which is known to induce the operation of the reverse mode of NCX, leading to an increase in [Ca^2+^]_i_ and a positive inotropic effect. Mitochondrial Ca^2+^ (mCa^2+^) uptake through the calcium uniporter (CaU) is in part dependent on the Ca^2+^ gradient between the cytosol and the mitochondrial matrix (Camara et al., 2010). Thus, it seems evident that the cytosolic Ca^2+^ increase, following the activation of NHE-1 and NBC, may lead to an increase in mCa^2+^. Mitochondrial Ca^2+^ loading regulates cellular respiration and mediates cell death (Camara et al., 2010). Calcium, through the activation of the CaMKII, was described as one of the main activators of NOX (Nishio et al., 2012) and mitochondrial ROS production (Song et al., 2011), creating a positive feed-back (feed-back 3, Figure 2) by which the ROS pathways acquire a central role in cell physiology (Trebak et al., 2010; Gul et al., 2012).

It has been described that the NHE-1 blockers attenuate the mCa^2+^ overload, ROS production and mPTP opening induced by ouabain (Toda et al., 2007). These authors proposed two possible mechanisms: (a) the NHE-1 inhibition prevents the increase in [Na^+^]_i_ and subsequent [Ca^2+^]_i_, which reduces the driving force for mCa^2+^ uptake, and (b) NHE-1 inhibition might indirectly activate the mitoK_ATP_ channel (the protection induced by NHE-1 blockers is prevented with the mitoK_ATP_ blocker 5-HD). In addition, it has been demonstrated that NHE-1 inhibition prevents mPTP opening during the first minutes after reperfusion, leading to an improvement of mitochondrial function as well as an attenuation of pro-apoptotic factors. In this work, the authors discussed several possibilities for the NHE-1-inhibition-induced protection, being the most important the attenuation of [Ca^2+^]_i_ overload and the delay of pH_i_ recovery during reperfusion (Javadov et al., 2008). Moreover, Garciarena et al. (2008) working on isolated mitochondria, showed that the NHE-1 inhibitors modulate mitochondrial ROS production via a direct mitochondrial action. However, the site of action has not been elucidated. Nevertheless, it is important to point out again that the careful regulation of ROS production, which involves the modulation of calcium handling, represents a crucial process in myocardial intracellular signaling (Figure 2).

## Mitochondrial ROS and cardiac pathology

When the cells are exposed to the same stimuli for long periods of time, they begin to lose their equilibrium, and in this scenario ROS and Ca^2+^ might represent dangerous molecules, leading to arrhythmias and cardiac hypertrophy (Terentyev et al., 2008; Zhao et al., 2011; Maulik and Kumar, 2012). In this regard, high mCa^2+^ impairs ATP synthesis leading to a loss in ion homeostasis, opening of mPTP and matrix swelling. The irreversible mPTP opening is associated with release of cytochrome C and more ROS production, resulting in a harmful vicious cycle of further amplification of ROS production, mCa^2+^ overload and irreversible cell damage, which lead to cell death (Camara et al., 2010). On the other hand, several investigations have demonstrated that a low increase in matrix ROS is sufficient to trigger brief, stochastic openings of mPTP, perhaps through reversible thiol oxidation (Wang et al., 2008). Moreover, these transient brief openings of mPTP have been involved as a “physiological valve”, alleviating mCa^2+^ overload and providing protection against cellular injury (Smaili and Russell, 1999; Kindler et al., 2003).

There are several evidences that involve the participation of ROS produced by NOX in different models of heart failure with RAAS activation (Sorescu and Griendling, 2002; Guo et al., 2006). Since NOX-produced ROS can be amplified by ROS generated by mitochondria during the ROS-induced-ROS release mechanism, this process could be also involved in the development of cardiac hypertrophy and the transition to heart failure. Indeed, it was reported that mice that overexpress catalase (antioxidant enzyme that degrades H_2_O_2_) targeted to mitochondria are resistant to cardiac hypertrophy, fibrosis and mitochondrial damage induced by Ang II as well as heart failure induced by overexpression of Gαq (Dai et al., 2011). In addition, Ang II-induced mitochondrial ROS are implicated in the development of apoptosis (Choudhary et al., 2008). Thus, breaking the ROS vicious cycle within mitochondria by antioxidants specifically targeted to this organelle would be effective to attenuate both cardiac hypertrophy and failure.

It was recently demonstrated that Ang II binds to AT_1_ and AT_2_ receptors localized in the mitochondrial inner membrane (mAT_1_ and mAT_2_) (Abadir et al., 2011). The authors of this study proposed an interesting model, where they associated the subtype of Ang II receptor and the type of ROS generated by mitochondria. In young animals, the activation of mAT_2_ induced protective mitochondrial NO generation. However, this protection disappeared with aging, possibly, due to harmful ROS producing an increased expression of mAT_1_. A similar speculation of mAT_1_ and mAT_2_ remodeling with aging could be done for cardiovascular diseases. In contrast, Doughan et al. (2008) demonstrated that Ang II does not exert any effect on isolated mitochondria. Moreover, it will be necessary to elucidate how Ang II gains access to the intracellular space, either by internalization or by local synthesis (Inagami, 2011). The relevance of this intracellular action of Ang II on mitochondrial ROS production remains to be studied.

## Final notes and perspectives

The main objective of this review was to emphasize the participation of mitochondria in the signaling pathways of RAAS. As we have shown, almost all the effects of RAAS involve the production of ROS, and the main source of them appears to be mitochondria. In summary, we attempted to call attention to the central role of cardiomyocyte mitochondria as the sites where the cellular signaling mediated by ROS converge.

Mitochondria have been mainly thought as the organelles that generate energy. However, in the last years, we have learned that mitochondria are much more than only an “ATP-plant production”. They are involved in cellular ion homeostasis, oxidative stress and cell survival or death. The main role of mitochondria seems to be the supervisor of cell functions. Mitochondria are the main source of ROS and, more important, they can interact with others sources of ROS in order to amplify the signal. Moreover, ROS regulate calcium signaling, which can also act as second messenger and further stimulate ROS production, creating a cycle, capable of being regulated at many steps (Figure 2).

As commented above, in the past ROS were thought exclusively as deleterious molecules. However, recent studies show that they appear to be very important second messengers that mediate different intracellular pathways. What has changed? The molecules, the stimuli and the sources are the same. Perhaps, we should believe that we are just beginning to uncover how the cells can use harmful reactive species for their own benefit.

Finally, we would like to highlight that mitochondrial ROS signaling may be an attractive target for therapeutic drugs in order to suppress RAAS signals. However, due to the complexity of the mechanisms involved, this idea requires careful evaluation, since targeting the wrong site at the wrong moment could not only worsen the disease, but could also suppress important physiological signaling pathways.

### Conflict of interest statement

The authors declare that the research was conducted in the absence of any commercial or financial relationships that could be construed as a potential conflict of interest.
